# Change of Firm

**Published:** 1869-11

**Authors:** 


*Change of Firm.*—The Phila. Dental Manufacturing Co., successors to Rubencame, Stockton & Duff, has passed into the hands of Messrs. Rubencame & Barker, by which title the firm is now designated.

